# Socioeconomic Status, Time Scarcity and Well-Being in Retirement

**DOI:** 10.1093/geroni/igab046.3289

**Published:** 2021-12-17

**Authors:** Boroka Bo

**Affiliations:** University of California, Berkeley, Berkeley, California, United States

## Abstract

We tend to think of retirement as a great equalizer when it comes to relief from the pernicious time scarcity characterizing the lives of many individuals in the labor force. Puzzlingly, this is not entirely the case. Using data from the MTUS (N=15,390) in combination with long-term participant observation (980 hours) and in-depth interviews (N=53), I show that socioeconomic characteristics are important determinants of retiree time scarcity. Neighborhood disadvantage gets under the skin via time exchanges that are forged by both neighborhood and peer network characteristics. The SES-based ‘time projects of surviving and thriving’ undergirding the experience of time scarcity lead to divergent strategies of action and differing consequences for well-being. For the advantaged, the experience of time scarcity is protective for well-being in later life, as it emerges from the ‘work of thriving’ and managing a relative abundance of choices. For the disadvantaged, the later life experience of time scarcity is shaped by cumulative inequality, further exacerbating inequalities in well-being. The final section of the article offers an analysis and interpretation of these results, putting retiree time scarcity in conversation with the broader literature on socioeconomic status and well-being.

